# Acupuncture for the treatment of trigeminal neuralgia: A protocol for the systematic review of randomized controlled trials

**DOI:** 10.1097/MD.0000000000010108

**Published:** 2018-03-16

**Authors:** Jong-In Kim, Hee-Jung Kim, Jung Ju Lee, Ji Hee Jun, Tae-Young Choi, Myeong Soo Lee

**Affiliations:** a Department of Clinical Korean Medicine, Graduate School, Kyung Hee University, Seoul; b Graduate School of East-West Medical Science, Kyung Hee University, Yongin; c Clinical Research Division, Korea Institute of Oriental Medicine, Daejeon, Republic of Korea.

**Keywords:** acupuncture, protocol, systematic review, trigeminal neuralgia

## Abstract

**Background:**

The ability of acupuncture to successfully control pain has been reported in the past. However, currently no systematic reviews exist regarding the effect of acupuncture on trigeminal neuralgia (TN). This proposed review aims to evaluate the current evidence on the efficacy of acupuncture for the management of pain in TN.

**Methods:**

A total of 11 databases were searched from their inception. These include MEDLINE, AMED, EMBASE, the Cochrane Library, 6 Korean medical databases, and 1 Chinese Medical Database. Study selection, data extraction, and assessment were performed independently by 2 researchers. Risk of bias was assessed via the Cochrane risk of bias assessment tool.

**Ethics and dissemination:**

Ethical approval is not required, given that this protocol is for a systematic review. The systematic review will be published in a peer-reviewed journal and disseminated both electronically and in print. The review was updated to inform and guide healthcare practice and policy.

**Trial registration number:**

PROSPERO 2018 CRD42018087594.

## 1 Introduction

### 1.1 Description of the condition

Trigeminal neuralgia (TN) is a type of severe chronic pain characterized by brief electric shock-like pains in 1 or more divisions of the trigeminal nerve.^[1]^ The frequency, duration, and severity of these painful attacks gradually increase, and they often become resistant to medication.^[2]^ Therefore, neuralgia becomes chronic and can severely affect the quality of life and cause cognitive disturbances, such as anxiety and depression, in the majority of patients with TN.^[3]^ The annual incidence of TN is between 4 and 27 per 100,000, affecting females more often than males. The prevalence increases at >50 years of age, and among young people it is often related to multiple sclerosis.^[4]^

### 1.2 Description of the intervention

Acupuncture, which originated in China more than 3000 years ago, has spread to over 160 countries and regions; it is used to treat various disorders such as pain and neurologic conditions, women's health, psychiatric disorders, cancer, and functional bowel disorders.^[5]^ In particular, the acupuncture-induced analgesic effect has been used widely to alleviate various pain conditions. A recent meta-analysis of individual patient data from a total of 20,827 patients participating in 39 trials showed that acupuncture is effective for controlling chronic pain and that treatment effects persist over time.^[6]^

### 1.3 How the intervention might work

The mechanism of action underlying the effects of acupuncture on controlling pain, so-called acupuncture analgesia, has been explored with regard to both peripheral and central mechanisms. Insertion of an acupuncture needle into an acupuncture point produces de-qi sensation, which excites all afferent fibers of the muscle tissue.^[7,8]^

Mechanical stimulation during acupuncture seems to pass signals via the sensory ganglia to the spinal cord and via interneurons to modulate the activity of motor neurons in the brain stem network, resulting in activation of various opioid receptors, which induce analgesic effects through descending inhibition in the supraspinal CNS region.^[7,9]^ The analgesic effect of acupuncture-related endogenous opioid peptides is prolonged.^[10]^ In addition, acupuncture also has an immediate analgesic effect. One possible mechanism of the immediate analgesia is the pathway termed diffuse noxious inhibitory controls (DNIC).^[11]^ According to DNIC, a noxious stimulus can induce immediate suppression of pain transmission in neurons of the trigeminal caudalis and /or the spinal horn.^[12]^

### 1.4 Why the intervention is important to this review

Anti-epileptic drugs, including carbamazepine, are the first-line treatment options for TN, but they often fail to relieve pain and/or produce significant side effects such as cognitive disturbances, memory loss, and bone marrow suppression.^[13,14]^ Among surgical interventions, microvascular decompression surgery may represent an effective method for curing TN.^[15]^ However, it is limited to neurovascular conflict in vascular compression of the trigeminal nerve root in young adults or healthy elderly and about half of the cases experience recurrent pain.^[2,16]^ Therefore, acupuncture may be an option for controlling pain in TN.

### 1.5 Objective

This proposed review aims to systematically evaluate the evidence on the safety and effectiveness of acupuncture for controlling pain in patients with TN from randomized controlled trials (RCTs).

## 2 Methods

### 2.1 Study registration

This protocol has been registered in PROSPERO 2018 CRD42018087594 http://(www.crd.york.ac.uk/PROSPERO/display_record.php?ID=CRD42018087594).

### 2.2 Criteria for considering studies for this review

#### 2.2.1 Types of studies

Prospective RCTs were included. We excluded observational, cohort, case-control, case series, qualitative studies, uncontrolled trials, and laboratory studies. No language restrictions were imposed.

#### 2.2.2 Types of participants

We included patients with TN regardless of age, sex, and origin of countries.

#### 2.2.3 Types of interventions and controls

Studies that have evaluated any type of invasive acupuncture with or without electrical stimulation were included. Studies investigating other methods of stimulating acupuncture points without needle insertion (acupressure, pressed studs, laser stimulation, etc.) were included but analyzed separately. Control interventions include treatments such as general conventional care (drugs, botulism toxin, etc.), sham treatment (interventions mimicking ‘true’ acupuncture/true treatment but deviating in at least 1 aspect considered important by acupuncture theory, such as skin penetration or correct point location), or waiting list care. We also included trials that compare acupuncture plus another active treatment with the same other active treatment alone. We excluded RCTs in which 1 type of acupuncture compared with a different type of acupuncture.

#### 2.2.4 Type of outcome measures

##### 2.2.4.1 Primary outcomes

(1) Severity of pain (measured with Visual Analogue Scale (VAS), numerical rating scale (NRS)), or other measurement tools for pain
(2) Frequency of attacks

##### 2.2.4.2 Secondary outcomes

(1) Total treatment efficacy: number of patients whose TN has improved
(2) Quality of life: measured using a validated questionnaire
(3) Adverse events (AEs)

### 2.3 Search method for identifying the studies

#### 2.3.1 Electronic searches

Electronic databases include MEDLINE, EMBASE, the Cochrane Central Register of Controlled Trials (CENTRAL), AMED, 6 Korean databases (KoreaMed, the Korean Traditional Knowledge Portal, Oriental Medicine Advanced Searching Integrated System (OASIS), DBpia, the Research Information Service System (RISS), and the Korean Studies Information Service System), and 1 Chinese database (China National Knowledge Infrastructure (CNKI)). Articles identified through reference lists of the included studies and relevant systematic reviews were considered for inclusion based on their title.

#### 2.3.2 Search of other resources

The authors scanned the reference lists of selected studies to retrieve additional studies. In addition, authors searched the WHO International Clinical Trials Registry Platform (ICTRP) (http://apps.who.int/trialsearch/), ClinicalTrials.gov registry (http://clinicaltrials.gov/) and other relevant trial registries.

#### 2.3.3 Search strategy

Our search strategy includes main keywords such as ‘acupuncture,’ ‘trigeminal neuralgia,’ ‘facial pain,’ and ‘idiopathic facial pain’ in English, Chinese, and Korean.

### 2.4 Data collection, extraction and assessment

#### 2.4.1 Selection of studies

Two reviewers (JIK and JHJ) independently screened the titles and abstracts of studies found in the searches and performed study selection, recorded their decisions according to predefined criteria. Another reviewer (MSL) resolved disagreements in study selection. Study selection was documented and summarized in a Preferred Reporting Items for Systematic Reviews and Meta-Analysis (PRISMA)-compliant flow chart (http://www.prisma-statement.org).

#### 2.4.2 Data extraction

All articles were read by 2 independent reviewers (JIK and HJK) who extracted data from the articles according to predefined criteria. The extracted data include the author name(s), year of publication, country, sample size, age and sex of study participants, acupuncture intervention, control intervention, main outcomes, and adverse effects. The extracted data were tabulated for future analysis. The authors used the Grading of Recommendations Assessment, Development and Evaluation (GRADE) software to determine the quality of evidence based on the Cochrane Handbook for Systematic Reviews of Interventions to create a Summary of Findings table.^[17]^ Details regarding the acupuncture method and control interventions were extracted based on the revised Standards for Reporting Interventions in Clinical Trials of Acupuncture (STRICTA).^[18]^ When reported data were insufficient or unclear, an author contacted the first author or corresponding authors by E-mail or telephone to request missing data or clarification of data.

#### 2.4.3 Assessment of risk of bias

Quality assessment was performed using the tool for assessing risk of bias from the Cochrane Handbook for Systematic Reviews of Interventions.^[19]^ The following characteristics were assessed.

(1) random sequence generation

(2) allocation concealment

(3) blinding of participants and personnel

(4) blinding of outcome assessment

(5) incomplete outcome data

(6) selective outcome reporting

(7) other sources of bias (we will evaluate baseline imbalance).

In this review L, U, and H as keys for these evaluations are used, where L (low) indicates a low risk of bias, U (unclear) indicates that the risk of bias is uncertain, and H (high) indicates a high risk of bias. Disagreements were resolved by discussion among all reviewers. Information regarding the risk of bias assessment for the included studies are summarized in table form, and the results and implications are critically discussed.

### 2.5 Data analysis

All statistical analyses were conducted using the Cochrane Collaboration's software Review Manager (RevMan), v.5.3 for Windows (The Nordic Cochrane Center, Copenhagen, Denmark). Differences between the intervention and control groups were also assessed. In the analysis of clinical efficacy, categorical data were assessed in terms of risk ratios and continuous data were assessed in terms of mean difference (MD). Categorical and continuous variables were expressed as efficacy values with 95% confidence intervals (CIs). In cases of outcome variables with different scales, standardized MD was used instead of weighted MD. When we detected heterogeneity (defined by results of tests of heterogeneity that indicate *P* < 0.1 via chi-square tests and Higgins *I*^2^ ≥ 50%), subgroup analyses were performed to find the cause of clinical heterogeneity. A random effects model was used to assess combined effect sizes from efficacy variables because substantial clinical heterogeneity was expected across the included studies based on the diversity of interventions, study designs, and other conditions. Publication bias were assessed using funnel plots and Egger's regression method.^[20]^ When missing data were detected, we requested any missing or incomplete information from the investigators of the original study.

Subgroup analysis was conducted according to different control interventions (acupuncture vs sham acupuncture; acupuncture vs conventional intervention; acupuncture combined with conventional intervention vs conventional intervention), and type of stimulation (manual vs. electric), Where appropriate, sensitivity analyses were performed to evaluate the robustness of the meta-analysis results.^[21]^

### 2.6 Ethics and dissemination

Ethical approval was not required, given that this protocol is for a systematic review. The findings of this review are disseminated widely through peer-reviewed publications and conference presentations.

## 3 Discussion

Until now, only 1 systematic review on the use of acupuncture for controlling pain in TN has been published.^[22]^ However, this review is outdated, and it did not follow the formal recommendations for a systematic review. The results of this systematic review evaluating the evidence on the safety and effectiveness of acupuncture for treating pain in TN from RCTs may be utilized by clinicians for managing pain in TN.

## References

[1] Cruccu G Gronseth G Alksne J AAN-EFNS guidelines on trigeminal neuralgia management. Eur J Neurol 2008;15:1013–28. 18721143 10.1111/j.1468-1331.2008.02185.x

[2] Spina A Mortini P Alemanno F Trigeminal neuralgia: toward a multimodal approach. World Neurosurg 2017;103:220–30. 28377244 10.1016/j.wneu.2017.03.126

[3] Peker S Sirin A Primary trigeminal neuralgia and the role of pars oralis of the spinal trigeminal nucleus. Med Hypotheses 2017;100:15–8. 28236840 10.1016/j.mehy.2017.01.008

[4] Hall GC Carroll D Parry D Epidemiology and treatment of neuropathic pain: the UK primary care perspective. Pain 2006;122:156–62. 16545908 10.1016/j.pain.2006.01.030

[5] Schnyer R Lao L Hammerschlag R Society for Acupuncture Research: 2007 conference report: “The status and future of acupuncture research: 10 years post-NIH Consensus Conference”. J Altern Complement Med 2008;14:859–60. 18803494 10.1089/acm.2008.SAR-2 PMC3155094

[6] Vickers AJ Vertosick EA Lewith G Acupuncture for chronic pain: update of an individual patient data meta-analysis. J Pain 2017. 10.1016/j.jpain.2017.11.005 PMC5927830 29198932

[7] Zhao ZQ Neural mechanism underlying acupuncture analgesia. Prog Neurobiol 2008;85:355–75. 18582529 10.1016/j.pneurobio.2008.05.004

[8] Yu JS Zeng BY Hsieh CL Acupuncture stimulation and neuroendocrine regulation. Int Rev Neurobiol 2013;111:125–40. 24215920 10.1016/B978-0-12-411545-3.00006-7

[9] Noguchi E Acupuncture regulates gut motility and secretion via nerve reflexes. Auton Neurosci 2010;156:15–8. 20663717 10.1016/j.autneu.2010.06.010

[10] Pomeranz B Chiu D Naloxone blockade of acupuncture analgesia: endorphin implicated. Life Sci 1976;19:1757–62. 187888 10.1016/0024-3205(76)90084-9

[11] Kawakita K Okada K Acupuncture therapy: mechanism of action, efficacy, and safety: a potential intervention for psychogenic disorders? Biopsychosoc Med 2014;8:4. 24444292 10.1186/1751-0759-8-4 PMC3996195

[12] Le Bars D Dickenson AH Besson JM Diffuse noxious inhibitory controls (DNIC). I. Effects on dorsal horn convergent neurones in the rat. Pain 1979;6:283–304. 460935 10.1016/0304-3959(79)90049-6

[13] Tentolouris-Piperas V Lee G Reading J Adverse effects of anti-epileptics in trigeminal neuralgiform pain. Acta Neurol Scand 2018. 10.1111/ane.12901 29377062

[14] Di Stefano G Truini A Pharmacological treatment of trigeminal neuralgia. Expert Rev Neurother 2017;17:1003–11. 28829200 10.1080/14737175.2017.1370375

[15] Bick SKB Eskandar EN Surgical treatment of trigeminal neuralgia. Neurosurg Clin N Am 2017;28:429–38. 28600016 10.1016/j.nec.2017.02.009

[16] Feller L Khammissa RAG Fourie J Postherpetic neuralgia and trigeminal neuralgia. Pain Res Treat 2017;2017:1681765. 29359044 10.1155/2017/1681765 PMC5735631

[17] Atkins D Best D Briss PA Grading quality of evidence and strength of recommendations. BMJ 2004;328:1490. 15205295 10.1136/bmj.328.7454.1490 PMC428525

[18] MacPherson H Altman DG Hammerschlag R Revised STandards for Reporting Interventions in Clinical Trials of Acupuncture (STRICTA): extending the CONSORT statement. J Altern Complement Med 2010;16:ST1–4. 20954957 10.1089/acm.2010.1610

[19] Higgins J Altman D Sterne J Chapter 8: assessing risk of bias in included studies. In: Higgins JPT, Green S. eds Cochrane handbook for systematic reviews of interventions version The Cochrane collaboration updated March 2011.

[20] Egger M Davey Smith G Schneider M Bias in meta-analysis detected by a simple, graphical test. BMJ 1997;315:629–34. 9310563 10.1136/bmj.315.7109.629 PMC2127453

[21] Hewitt C Hahn S Torgerson DJ Adequacy and reporting of allocation concealment: review of recent trials published in four general medical journals. BMJ 2005;330:1057–8. 15760970 10.1136/bmj.38413.576713.AE PMC557225

[22] Liu H Li H Xu M A systematic review on acupuncture for trigeminal neuralgia. Altern Ther Health Med 2010;16:30–5. 21280460

